# Syphilitic Deformity of the Teeth

**Published:** 1882-08

**Authors:** 


### ARTICLE XI

### *Syphilitic Deformity of the Teeth.*

Before the Odontological Society of Great Britan Mr. Ackery showed two cases of unilateral syphilitic deformity of the upper central incisiors; in each case the left central showed the typical notch while the right was normal, which posssessed some interesl as an usual occurrence, since since nearly all syphilitic manifestations are bilateral. Mr. Coleman presented a model of a case in which there were two supernumerary centrals of distinctly syphletic type, while the proper centrals, which were coming down within the arch, were well formed. The patient presented other evidences of syphilitic taint. Before a meeting of the same Society (*British Medical Journal*) Dr. B. W. Richardson read a paper on "The Causes of Dental Caries, Constitutional and Local." For some years he has kept a record of the condition of the teeth of all patients that came before him. He found that, of over four thousand persons, of both sexes and all ages, over eighty per cent. were affected more or less severely with dental caries; while it was rare to meet with a person in whom both sets of teeth were altogether free from the disease. The two general causes which he believes to be chiefly responsible for this condition of affairs are hereditary syphilis and dyspepsia. With regard to the first, he quoted the statements of Professor Gross and Dr. Holland, respectlng the prportion of the adult population of the United States and Great Britain, respectively, who acquire the primary disease, estimated in each case at about one in eight. Contracted in adult life syphilis does not materially affect the teeth; but the hereditary constitution bequeathed by it is undoubtedly indicated in the next generation by disease of the teeth, and by a constitutional condition in which caries is readily developed. It is hard to say whether dyspepsia should be placed before or after syphilis in point of importance.—*Medical Times and Gazette.*

---

### EDITORIAL, ETC.

